# How weeds emerge: a taxonomic and trait-based examination using United States data

**DOI:** 10.1111/nph.12698

**Published:** 2014-02-04

**Authors:** Adam Kuester, Jeffrey K Conner, Theresa Culley, Regina S Baucom

**Affiliations:** 1 Department of Ecology and Evolutionary Biology, University of Michigan2059 Kraus Natural Science Building, Ann Arbor, MI, 48109, USA; 2 Kellogg Biological Station and Department of Plant Biology, Michigan State University3700 East Gull Lake Drive, Hickory Corners, MI, 49060, USA; 3 Department of Biological Sciences, University of Cincinnati724 Rieveschl Hall, Cincinnati, OH, 45221, USA

**Keywords:** herbicide resistance, introduced plant, taxonomic selectivity, weed

## Abstract

1. Weeds can cause great economic and ecological harm to ecosystems. Despite their importance, comparisons of the taxonomy and traits of successful weeds often focus on a few specific comparisons – for example, introduced versus native weeds.
2. We used publicly available inventories of US plant species to make comprehensive comparisons of the factors that underlie weediness. We quantitatively examined taxonomy to determine if certain genera are overrepresented by introduced, weedy or herbicide-resistant species, and we compared phenotypic traits of weeds to those of nonweeds, whether introduced or native.
3. We uncovered genera that have more weeds and introduced species than expected by chance and plant families that have more herbicide-resistant species than expected by chance. Certain traits, generally related to fast reproduction, were more likely to be associated with weedy plants regardless of species’ origins. We also found stress tolerance traits associated with either native or introduced weeds compared with native or introduced nonweeds. Weeds and introduced species have significantly smaller genomes than nonweeds and native species.
4. These results support trends for weedy plants reported from other floras, suggest that native and introduced weeds have different stress adaptations, and provide a comprehensive survey of trends across weeds within the USA.

## Introduction

Weedy plants are considered troublesome because they are prolific and highly adaptable and often persist in large numbers in areas where they are not wanted (Quamman, 1998; Radosevich *et al*., 2007). They can cause severe economic impact (Pimentel *et al*., 2000, 2005) as a result, at least in part, of their ability to adapt to regimes of human-mediated selection (De Wet & Harlan, 1975) – for example, there are now > 200 cases of evolved herbicide-resistant weedy plant species following only *c*. 60 yr of herbicide use (Heap, 2013). The term ‘weed’ in the most general sense describes ‘a plant growing out of place’ (Radosevich *et al*., 2007), and thus a variety of problematic plants – whether native or introduced, and whether inhabiting wild, agricultural or other managed areas – are often considered as weeds even though there may be distinct and interesting differences among subgroups (Daehler, 1998). The term ‘invasive’ is often used to indicate problematic plants that can successfully establish and spread following introduction into novel, often nonmanaged areas (Radosevich *et al*., 2007). While invasives are considered weeds in the broadest sense of the term, some authors prefer to use the designation ‘weed’ only for problematic plants in agriculture, and reserve the term ‘invasive’ to indicate problematic plants growing in nonmanaged or wild areas (Randall, 1997; Radosevich *et al*., 2007; Ellstrand *et al*., 2010); problematic plants in nonmanaged areas are also called ‘wildland weeds’ (Randall, 1997).

In addition to the lack of a uniform terminology employed by researchers to delineate problematic plant species, understanding their emergence in any flora is often complex because of their diverse and unique histories. Are problematic weeds native and adapting to changing regimes of human disturbance, or, after introduction and following naturalization, did they adapt to exploit a novel resource? Are they problematic because they are herbicide resistant? A rich literature has addressed the central question of why some species, and not others, are problematic or weedy. The search for the determinants of weediness includes comparisons of species traits (Baker, 1965; Keeler, 1989; Williamson, 1993; Rejmánek & Richardson, 1996; Radford & Cousens, 2000; Sutherland, 2004; van Kleunen *et al*., 2010; Moravcova *et al*., 2010), introduction histories and residence times (reviewed in Kolar & Lodge, 2001; Pyšek 2005; Pemberton & Liu, 2009), and ecological and evolutionary processes (DeWalt *et al*., 2004; Bossdorf *et al*., 2005; Schierenbeck & Ellstrand, 2009); what these different types of study have in common is that they are all performed in a comparative framework (van Kleunen *et al*., 2010) – that is, by comparing weeds to nonweeds or invasives to noninvasives.

**Figure 1:**
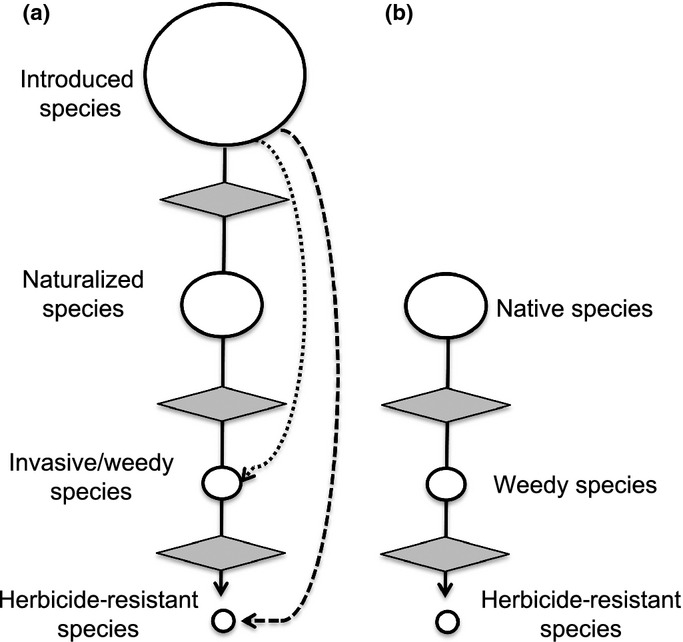
A simplified version of the problematic species continuum, adapted from Williamson (1996) and applied to (a) introduced and (b) native species (the sizes of the circles in (a) and (b) are not comparable). In (a), the circle represents the proportion of species that become naturalized, invasive, and herbicide resistant, and dashed and dotted lines depict the introduction of species that were herbicide resistant and invasive/weedy in their native area, respectively. In both (a) and (b), diamonds represent environmental filters as envisioned by Williamson (1996).

In the field of invasion ecology, a variety of conceptual frameworks have been proposed to understand the success of invasive species (van Kleunen *et al*., 2010; Richardson & Pyšek, 2012). Many frameworks incorporate the concept of ‘the continuum,’ or the different stages of biological invasion. Briefly, the continuum spans species that are introduced but not yet naturalized, those that are introduced and naturalized (i.e. able to reproduce without the aid of humans), and, finally, introduced species that are considered noxious (i.e. invasive/weedy; Fig.1). In Richardson *et al*.’s (2000) scheme, environmental barriers to growth and reproduction must be overcome for the species to evolve into an invasive (Booth *et al*., 2004); in the case of invasive plants, different characteristics, such as climate tolerance and/or certain reproductive traits, are hypothesized to be more important than others at different stages in the continuum (Williamson, 1993a,b, 1996). The general framework of the continuum, however, provides a useful structure with which to identify traits or characteristics of problematic plant species broadly – for example, by comparing traits of weeds to nonweeds, introduced to native species, and native weeds to native nonweeds, and the continuum’s more specific comparison of introduced plants that are considered ‘problematic’ or ‘weedy’ to plants that are introduced and not yet causing great harm to an ecosystem. Here, we co-opt the conceptual framework of the continuum to make a variety of comparisons between these groups of plants found within the USA (see Fig1).

Despite the utility of the invasive plant continuum, it is striking that it currently lacks mention of herbicide resistance, a global problem that plagues most weedy plant chemical control efforts (Gianessi, 2005) and leads to an expenditure of $US4bn per year (Pimentel *et al*., 2005). This perhaps results from the focus of the continuum on plants that are invasive of nonmanaged areas (i.e. as defined above), and a higher expectation of herbicide resistance in agricultural weeds rather than invasives, as many herbicides are not registered for use in natural areas (Smith *et al*., 2006). However, there are certainly cases of introduced invasives of natural areas that are herbicide resistant (e.g. *Sinapis arvensis*). Introduced species can become herbicide resistant from intense selection via herbicide application; it is likewise possible that species previously selected for herbicide resistance in their native ranges can be introduced into new areas – we show this possibility as an accelerated trajectory in Fig.1. In light of the global ubiquity of herbicide-resistant plants along with the economic stress imposed by resistant species, we suggest that herbicide resistance should be considered as another important step in the continuum and not simply as a characteristic of weedy plants. However, it is currently unknown if most herbicide-resistant species are native or introduced in the USA, and it is also unclear how many herbicide-resistant species are agricultural weeds versus natural area invaders. Addressing these gaps in our knowledge will help to determine where and how herbicide resistance may fit into the continuum concept.

The USA has a significant introduced and weedy species problem (Crall *et al*., 2006). Estimates of the number of introduced plants in the USA range between 3000 and 5000, which is a considerable number since there are *c*. 17 000 native plant species (Pimentel *et al*., 2005). Furthermore, the USA has more documented herbicide-resistant weeds than any other country (74 spp; Heap, 2013). Introduced plants can cause significant ecological impacts by displacing native plants, releasing novel pathogens, poisoning livestock and, in a few cases and at their worst, altering entire ecosystems (e.g. kudzu (*Pueraria lobata*), yellow star thistle (*Centaurea solstitralis*), and European cheatgrass (*Bromus tectorum*)) (DiTomaso, 2000; Randall, 2000; Forseth & Innis, 2004; Capinera, 2005; Prater & DeLucia, 2006; Vilà *et al*., 2011; Palumbo, 2012). Agricultural weeds can likewise poison livestock, bind to harvesting machinery, and compete with row crops to decrease the quantity and quality of US food production (Shaw, 1964; Williams, 1980; van Heemst, 1985). The influence of introduced and herbicide-resistant plants on both the ecological and economic health of US agriculture, tourism, and conservation is substantial; however, a comprehensive and broad analysis that delineates between ‘problematic’ and currently ‘benign’ introduced and native plant species in the US flora is absent.

To perform such an analysis, we used data from several publicly available databases to group problematic plant species – those considered either invasive of natural areas or agricultural weeds – in a ‘problematic plants’ category (similar to Pyšek *et al*., 1995) and determine what taxonomic and phenotypic differences exist among plants at differing stages of the continuum (i.e. by comparing introduced nonweeds/noninvasives to introduced weeds/invasives, and native weeds to native nonweeds; see Fig.1). For clarity and from this point forward, we refer to invasive and/or weedy species as ‘weedy,’ also in accordance with the designation used in the Composite List of Weeds (see Materials and Methods). We first used a taxonomic approach to determine if US plant genera are more or less likely to contain introduced, weedy, or herbicide-resistant members than would be expected by chance. We also tested for significant trait differences between weeds and nonweeds, within introduced and native species separately. Finally, we compared genome size and time of residency among our plant groups, as these factors have previously been linked to invasiveness and/or weediness status (Rejmánek & Richardson, 1996; Lavergne *et al*., 2009; Chen *et al*., 2010).

## Materials and Methods

### Data sets

**Figure 2:**
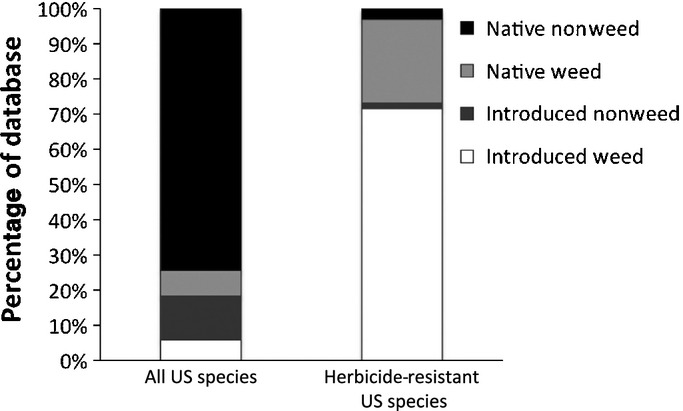
The percentage of species from our data set of all US (*n* = 19 180) and herbicide-resistant (*n* = 67) species categorized as introduced weeds, introduced nonweeds, native weeds, and native nonweeds.

Taxonomic information and introduction status (i.e. introduced (I) or native (N)) were obtained from the US Department of Agriculture (USDA) Plants Database (http://plants.usda.gov) and restricted to species found in the lower 48 states, excluding the flora of Hawaii and Alaska. In total, 19 180 species were present in our data set, representing 2829 genera and 270 plant families. We included synonyms of species within this database for reconciliation of taxa. Weeds, defined as troublesome plants in agriculture, horticulture, ornamental and natural areas (T. Miller, personal communication), were identified using a list from the 2010 Composite List of Weeds compiled by the Standardized Plant Names subcommittee from the Weed Science Society of America (WSSA) database (http://www.wssa.net; downloaded February 2010). This list consists of 3488 species, representing 1037 genera and 172 families, and is updated periodically to include species on state noxious weed lists (T. Miller, personal communication). Because the state noxious weed lists often contain species that are ‘invasive’ – that is, invaders of natural areas – our ‘weedy’ designation does not explicitly distinguish between agricultural weeds and invasive plant species (for comparison of the two, see Daehler, 1998). Of the 3488 species in the WSSA database, 2513 were present and/or able to be reconciled with taxa in the USDA Plant Database, which represent 935 genera (average of 2 species per genus). Of the 977 species that we could not match between the two data sets, 142 were duplicates (subspecies or varieties) in the WSSA database, 783 species were removed because the USDA data set had missing data regarding the location of the species (no data in the ‘floristic area’ data field), 45 species were found outside of the lower 48 states, and the rest were nomenclature inconsistencies between the two databases. The percentages of US species categorized as introduced weeds (IWs), introduced nonweeds (INWs), native weeds (NaWs), and native nonweeds (NaNWs) are presented in Fig.2.

We identified species in the database that were herbicide resistant in the USA from http://www.weedscience.org, which defines resistance as ‘an inherited ability of a plant to survive and reproduce following exposure to a dose of herbicide normally lethal to the wild type’ (Heap, 2013). Sixty-seven of the 74 species identified as herbicide resistant by the WSSA were present in the USDA database for the lower 48 states. Four of the seven species not present in the USDA database were not recognized in the Plant List of accepted names (http://www.plantlist.org). We further performed literature searches for the 67 herbicide-resistant species to determine whether they were agricultural weeds or invasive species of natural areas within the USA.

Genome size estimates were obtained from the Kew Plant database (http://data.kew.org/cvalues/). In total, we were able to locate genome size information for 3941 species in the data set, as follows: INW, 1354; IW, 1283; NaNW, 915; NaW, 389.

To compare the introduction times of IWs and introduced nonweeds, we randomly selected 100 species per group (INW and IW) from the USDA data set and recorded the first reported herbarium specimen present in the Global Biodiversity Information Facility (GBIF; http://data.gbif.org/). We restricted our search to US herbariums and searched for the earliest recorded specimen; we realize that this is an underestimate of time since introduction. If a species from our data set was not present in the GBIF, we replaced that species with another randomly selected species. We searched for 100 randomly chosen species of each designation rather than the full database as the searches proved relatively time-consuming. A summary of the sample sizes utilized in each analysis is presented in Supporting Information Table S1.

### Taxonomic analysis

We used a taxonomic enrichment analysis described by Lockwood (1999) to determine if particular genera or families are overrepresented by more weedy, introduced, or herbicide-resistant species than would be expected by chance. Analyses were performed for weediness and introduction status at the level of the genus, whereas the analysis of herbicide resistance was performed at both the genus and family levels. Herbicide resistance was performed at the family level even though sample sizes were comparatively low (i.e. weedy, *n* = 2513; introduced, *n* = 3524; herbicide resistant, *n* = 67). We used the binomial distribution to generate a random expectation of the number of species per genus considered weedy, introduced, or herbicide resistant (Bennett & Owens, 1997; Lockwood, 1999):

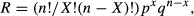
where *n* is the number of species within a genus or family in the database, *p* is the overall proportion of species that were considered weedy, introduced, or herbicide resistant, *q* is the overall proportion of species not classified as weedy, introduced or herbicide resistant, and *X* is the observed number of weedy, introduced, or herbicide-resistant species within a genus or family. Each *R* was adjusted for multiple tests with a Bonferroni correction (Miller, 1981), and a significance level of α = 0.05. If a group (genus or family) had significantly more weedy, introduced, or herbicide-resistant species than expected by chance, it was regarded as being taxonomically enriched for these designations. We performed this test for introduced species regardless of their ‘weedy’ status and then again only for native species to determine if there is a taxonomic bias of weediness within native flora.

### Phenotypic characteristics defining weedy and introduced plants

We utilized the presence/absence of plant characteristics from the USDA Plants Database to test for differences in traits according to introduction status (introduced/native) and weediness status (weed/nonweed). To focus on traits previously considered to be weedy, we restricted our tests to the 12 (out of 30 in the USDA data set) plant characteristics most similar to those of Baker (1965) and Whitney & Gabler (2008). A total of 1777 species (636 genera) had the relevant trait data in the USDA database, of which 465 were native weeds (250 genera), 157 were introduced weeds (105 genera), 121 were introduced nonweeds (97 genera) and 1034 were nonweedy natives (401 genera). The following traits were examined: whether a species is annual, has rapid growth, exhibits CaCO_3_, salinity or shade tolerance, and exhibits high fruit abundance, fruits that persist on the plant, rapid seed spread, high seedling vigor, and fast vegetative spread. The traits are considered ‘high’ or ‘rapid’ for each species if they exhibit high or rapid values relative to other species with the same growth habit. High salinity tolerance refers to species that can tolerate (or exhibit < 10% reduction in plant growth in) > 8.0 dS m^−1^ of salt. Because high versus low CaCO_3_ tolerance was not defined in the character information page (http://plants.usda.gov/charinfo.html), we elected to analyze these terms as two separate characters. Tolerance to CaCO_3_ was of interest because wastelands, roadsides, and agricultural land are often high in CaCO_3_, and we hypothesized that weeds may differ from nonweeds, by being better able to survive in a novel niche compared to nonweeds, because of their ability to tolerate this compound. Traits within this database are recorded as binary data and, while we recognize that each trait could be measured as a continuous character, we were restricted to modeling only their presence/absence.

To determine if introduced or weedy species were more or less likely to exhibit each of the above traits, we performed logistic regressions modeling trait presence/absence (1/0) using a binomial distribution with introduction and weed status, and their interaction, as fixed effects using the glm function in the base stats package of R (http://www.R-project.org). We used the general logistic regression model: *P*(*Y* = 1) = 1/(1 + exp[−(*B*_0_ + *B*_1_*x*_1_ + *B*_2_*X*_2_ + … + *B*_p_*X*_p_)]) with a binomial distribution and the default logit canonical link function (Crawley 2013). We included the interaction between introduced status and weedy status to test whether the presence of each trait varied for introduced weeds and nonweeds differently than for native weeds and nonweeds. We then divided the data into introduced or native species and compared trait presence/absence between introduced weeds and introduced nonweeds (IWs versus INWs) and between native weeds and native nonweeds (NaWs versus NaNWs) to examine whether traits were more or less likely to occur in plant species at different stages of the continuum. We also compared the presence/absence of the traits between introduced and native weeds to determine if there are traits specific to either group of weeds.

Because trait information is present for a higher proportion of weeds than nonweeds relative to their total numbers in the database (33% NaW, 14% IW, 5% INW, and 7% NaNW), we performed a preliminary comparison of traits between groups after adjusting for sample size biases – from this analysis, we detected no difference in the proportion of a group possessing a specific trait when compared to results obtained from the entire data set. We thus present analyses using the entire data set. We analyzed each trait separately, as preliminary analyses indicated that 93% of the phi coefficients between the traits were < 0.3, which indicates that there was little or no association between traits. Three phi coefficients, those estimated between seedling vigor and growth rate, fruit abundance, and seed spread rate, were > 0.3 (see Table S2 for phi correlation coefficients between traits), but still < 0.5.

To determine if the genome size of US plant species differed according to introduction and/or weediness status, we performed an analysis of variance using the ‘lm’ option from the built-in stats package of R (http://www.R-project.org) using the log-transformed C-value (the amount of an organism’s nuclear DNA in the unreplicated gametic nucleus (Kew C-value database; http://data.kew.org/cvalues)) as the dependent variable and introduction status and weed status, and the interaction between the two, as independent variables. If more than one C-value was reported for a species, we used the log-transformed average C-value for the species as the dependent variable. We used a Kruskal–Wallis rank sum test to determine if there were significant genome size differences between native weeds and native nonweeds and between introduced weeds and introduced nonweeds. Finally, we determined if introduced weeds have been a part of the US flora longer than introduced nonweeds (using 100 randomly sampled species from each classification; INW versus IW) using a Wilcoxon nonparametric test, as the data did not meet the assumption of normality.

## Results

### Taxonomic representation of weedy and introduced plants

Overall, 935 (33.1%) of 2829 genera contained at least one weedy species. Fifteen genera within 12 families had a significantly higher proportion of weedy species than expected by chance (Table1). These weedy genera represent a diverse group of plant families that exhibit a wide range of ecological and phenotypic diversity. For example, these weeds have different growth habits (grasses, forbs, shrubs, and trees), are present in diverse habitats (xeric versus aquatic; e.g. *Acacia* and *Lemna*) and exhibit different pollination syndromes (wind versus animal pollination; e.g. *Amaranthus* and *Solanum*). Among the genera overrepresented for ‘weediness,’ compared with the total database, we found more grasses (31 versus 13% for weed species versus total) and nonwoody species (90 versus 82%, respectively), but similar representation of trees (2 versus 4%, respectively), and slightly fewer shrubs (8 versus 13%, respectively) and herbaceous species (59 versus 67%, respectively). Most of the weedy genera were eudicots (72%) and the remainder (28%) were monocots.

**Table 1:** The 15 genera (0.53%) out of the 2829 genera from the 2010 US Department of Agriculture (USDA) Plants Database significantly more likely to contain weedy species than expected by chance

Genus	Family	Growth form	No. of species in genus	No. of weedy species	Proportion weedy	*R*-value
*Polygonum*	Polygonaceae	Herb	70	24	0.34	3.60E-06
*Cyperus*	Cyperaceae	Graminoid	97	36	0.37	2.23E-09
*Ipomoea*	Convolvulaceae	Herb	49	19	0.39	4.73E-06
*Amaranthus*	Amaranthaceae	Herb	41	17	0.41	5.15E-06
*Solanum*	Solanaceae	Herb	64	27	0.41	3.26E-08
*Chenopodium*	Chenopodiaceae	Herb	45	20	0.41	1.59E-06
*Acacia*	Fabaceae	Tree	33	15	0.45	5.15E-06
*Rumex*	Polygonaceae	Herb	41	20	0.49	3.13E-08
*Centaurea*	Asteraceae	Herb	31	16	0.52	2.76E-07
*Urochloa*	Poaceae	Graminoid	20	12	0.60	9.27E-06
*Phalaris*	Poaceae	Graminoid	11	8	0.73	9.4E-06
*Brassica*	Brassicaceae	Herb	8	7	0.88	4.61E-06
*Malva*	Malvaceae	Herb	8	7	0.88	4.61E-06
*Lemna*	Lemnaceae	Herb	9	8	0.89	6.79E-07
*Salsola*	Amaranthaceae	Herb	6	6	1.00	5.06E-06

For each genus, the most common growth form, the total number of species in the database, the number of weedy species, the proportion of species that are weedy, and the probability of being weedy (*R*-value) are shown. The expected proportion of weedy species from the entire data set was 0.1309.

Further, we found that 1317 (46.6%) of 2829 genera in our data set of US plant species contained at least one introduced species, and 40 genera, representing 20 families, were significantly overrepresented by introduced members (Table2). These genera represent a range of growth habits (forbs, shrubs, and trees), habitat types (e.g. xeric to aquatic), and pollination biology (selfing, and animal and wind pollinated). Twenty-one of these 40 genera had no native species within the USA. For comparison, we present the genera that are overrepresented by both introduced and weedy species in Table3. These genera consisted of perennials and annuals and ranged in habit, with representatives of grasses, forbs, shrubs and trees.

**Table 2:** The 40 genera (1.41%) of the 2829 genera from the 2010 US Department of Agriculture (USDA) Plants Database that contained more introduced species than expected by chance

Genus	Family	Growth form	No. of species in genus	No. of introduced species	Proportion introduced	*R*-value
*Polygonum*	Polygonaceae	Herb	70	28	0.40	1.40E-05
*Solanum*	Solanaceae	Herb	64	28	0.44	1.89E-06
*Bromus*	Poaceae	Graminoid	49	23	0.47	3.58E-06
*Eragrostis*	Poaceae	Graminoid	49	24	0.49	8.73E-07
*Rosa*	Rosaceae	Shrub	55	28	0.51	4.02E-08
*Lonicera*	Caprifoliaceae	Shrub	37	19	0.51	4.82E-06
*Acacia*	Fabaceae	Shrub	33	18	0.55	2.83E-06
*Rumex*	Polygonaceae	Herb	41	23	0.56	6.30E-08
*Geranium*	Geraniaceae	Herb	32	20	0.63	3.83E-08
*Vicia*	Fabaceae	Herb	31	20	0.65	1.76E-08
*Cerastium*	Caryophyllaceae	Herb	18	12	0.67	8.17E-06
*Urochloa*	Poaceae	Graminoid	20	14	0.70	5.77E-07
*Crotalaria*	Fabaceae	Herb	17	12	0.71	3.34E-06
*Veronica*	Scrophulariaceae	Herb	28	21	0.75	1.02E-10
*Erodium*	Geraniaceae	Herb	12	10	0.83	1.94E-06
*Tragopogon*	Asteraceae	Herb	10	9	0.90	1.96E-06
*Sisymbrium*	Brassicaceae	Herb	10	9	0.90	1.96E-06
*Centaurea*	Asteraceae	Herb	31	28	0.90	6.19E-18
*Pennisetum*	Poaceae	Graminoid	14	13	0.93	3.13E-09
*Verbascum*	Scrophulariaceae	Herb	17	17	1.00	3.13E-13
*Medicago*	Fabaceae	Herb	16	16	1.00	1.70E-12
*Linaria*	Scrophulariaceae	Herb	14	14	1.00	5.03E-11
*Cotoneaster*	Rosaceae	Shrub	14	14	1.00	5.03E-11
*Phyllostachys*	Poaceae	Graminoid	12	12	1.00	1.49E-09
*Eucalyptus*	Myrtaceae	Tree	12	12	1.00	1.49E-09
*Pelargonium*	Geraniaceae	Shrub	11	11	1.00	8.10E-09
*Clerodendrum*	Verbenaceae	Shrub	11	11	1.00	8.10E-09
*Narcissus*	Liliaceae	Herb	10	10	1.00	4.41E-08
*Tamarix*	Tamaricaceae	Tree	9	9	1.00	2.40E-07
*Ligustrum*	Oleaceae	Shrub	9	9	1.00	2.40E-07
*Gypsophila*	Caryophyllaceae	Herb	9	9	1.00	2.40E-07
*Malva*	Malvaceae	Herb	8	8	1.00	1.30E-06
*Kalanchoe*	Crassulaceae	Herb	8	8	1.00	1.30E-06
*Jasminum*	Oleaceae	Shrub	8	8	1.00	1.30E-06
*Citrus*	Rutaceae	Tree	8	8	1.00	1.30E-06
*Brassica*	Brassicaceae	Herb	8	8	1.00	1.30E-06
*Genista*	Fabaceae	Herb	7	7	1.00	7.09E-06
*Echium*	Boraginaceae	Herb	7	7	1.00	7.09E-06
*Dianthus*	Caryophyllaceae	Herb	7	7	1.00	7.09E-06
*Avena*	Poaceae	Graminoid	7	7	1.00	7.09E-06

For each genus, the most common growth form, the total number of species in the database, the number of introduced species, the proportion of species that are introduced and the probability of being weedy (*R*-value) are shown. The expected proportion of introduced species from the entire data set was 0.1846.

**Table 3:** Seven of 2829 genera from the US Department of Agriculture (USDA) Plant Database that contained both more weedy and more introduced species than expected by chance

Genus	Family	No. in genus	No. weedy	Proportion weedy	No. introduced	Proportion introduced	No. of introduced weeds	Proportion of introduced weeds
*Polygonum*	Polygonaceae	70	24	0.34	28	0.40	14	0.20
*Solanum*	Solanaceae	64	27	0.42	28	0.44	16	0.25
*Acacia*	Fabaceae	33	15	0.45	18	0.55	9	0.27
*Rumex*	Polygonaceae	41	20	0.49	22	0.54	11	0.27
*Centaurea*	Asteraceae	31	16	0.52	28	0.90	16	0.52
*Urochloa*	Poaceae	20	12	0.60	14	0.70	8	0.40
*Brassica*	Brassicaceae	8	7	0.88	8	1.00	7	0.88
*Malva*	Malvaceae	8	7	0.88	8	1.00	7	0.88

For each genus, the total number of species in the database, the number of weedy species, the proportion of weedy species, the number of introduced species, the proportion of introduced species, the number of species considered introduced and weedy, and the proportion of species considered introduced and weedy are shown.

To determine if the presence of nonnative species influenced our assessment of which genera were overrepresented by weedy members, we removed introduced species and repeated the above taxonomic enrichment analysis. We then uncovered seven plant genera enriched for weedy members. Only three genera were shared with the analysis of the entire data set (*Amaranthus*, *Cyperus* and *Lemna*; Table4), suggesting that the results of the taxonomic enrichment analysis of weedy plants are driven, in large part, by introduced species. Many of the genera enriched for native weeds are represented by aquatic or semi-aquatic members (*Cyperus*, *Lemna*, *Hydrocotyle*, and *Bacopa*); another genus of note, *Toxicodendron*, contains both poison ivy (*Toxicodendron radicans*) and poison oak (*Toxicodendron diversilobum*).

**Table 4:** Seven of 2829 (0.28%) genera from the 2010 US Department of Agriculture (USDA) Plants Database significantly more likely to contain weeds than expected by chance, identified after the removal of introduced species from the data set

Genus	Family	No. of weedy species	No. of species in genus	Proportion weedy	*R*-value
*Quercus*	Fagaceae	22	190	0.12	1.00E-25
*Cyperus*	Cyperaceae	25	77	0.32	8.00E-05
*Bidens*	Asteraceae	11	25	0.44	4.00E-05
*Amaranthus*	Amaranthaceae	12	27	0.44	2.00E-05
*Lemna*	Lemnaceae	8	9	0.89	4.00E-08
*Bacopa*	Scrophulariaceae	6	6	1.00	5.00E-07
*Toxicodendron*	Anacardiaceae	5	5	1.00	6.00E-06

For each genus, the total number of species in the database, the number of weedy species, the proportion of species that are weedy, and the probability of being weedy (*R*-value) are shown.

In addition to the overrepresentation of weedy and introduced species within genera, we also uncovered genera that were underrepresented for weediness and introductions – 10 genera from nine families had fewer weedy species than expected by chance (Table S3), and 30 genera from 19 families had fewer introduced species than expected (Table S4).

### Phenotypic characteristics of weedy and introduced plants

We next performed a series of comparisons to test for traits that differ according to introduction and weed status. The traits we considered were similar to those identified by Baker (1965) and Whitney & Gabler (2008) as typifying weedy or invasive plants, but our analysis expands on this work by making three more specific pairwise comparisons – introduced nonweeds versus introduced weeds, native nonweeds versus native weeds, and native weeds versus introduced weeds.

**Figure 3:**
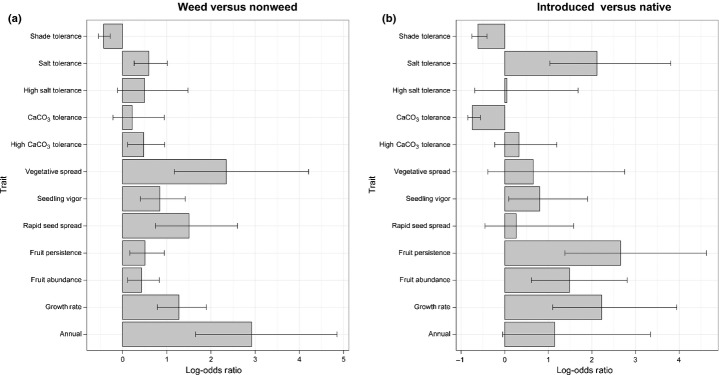
Comparison of traits related to growth, reproduction and tolerance of edaphic factors between (a) weeds and nonweeds and (b) introduced and native species. The common log-odds ratios and 95% confidence intervals (CIs) are shown. The height of each bar indicates the relative frequency of each trait, with values > 0 indicating that the frequency is higher among weeds in (a) and introduced species in (b).

**Figure 4:**
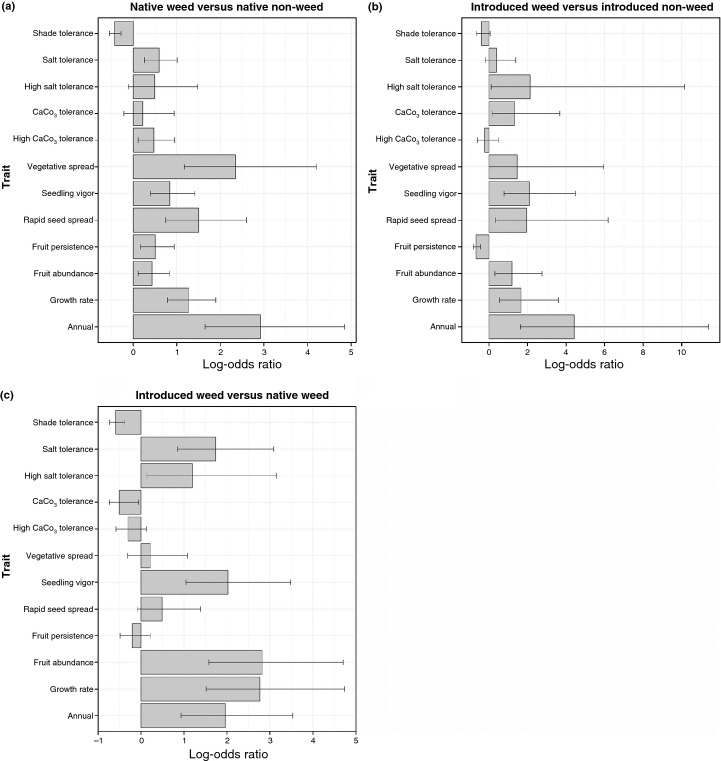
Comparison of traits related to growth, reproduction and tolerance of edaphic factors between (a) native weeds and native nonweeds, (b) introduced weeds and introduced nonweeds, and (c) native weeds and introduced weeds. The common log-odds ratios and 95% confidence intervals (CIs) are shown. The height of each bar indicates the frequency of each trait, with values > 0 indicating that the frequency is higher among native weeds versus native nonweeds in (a), introduced weeds versus introduced nonweeds in (b), and introduced weeds versus native weeds in (c).

Overall, we found that weedy plants, introduced and native combined, are more likely to be annual, exhibit a fast growth rate, and have high fruit abundance, fruits that persist on the plant, rapid seed spread, high seedling vigor, fast vegetative spread, and high CaCO_3_ and salt tolerance compared with nonweeds (Fig.3a; Table5); however, weedy species are less likely to be shade tolerant than nonweeds (Fig.3a; Table5). Our sample of introduced, naturalized species are more likely to exhibit many of these same traits when compared with native species – introduced species are more likely to be annual, exhibit a fast growth rate, and have high fruit abundance, fruits that persist on the plant, high seedling vigor, and high salt tolerance compared with native species (Fig.3b; Table5). Introduced species are less likely, however, to be CaCO_3_ and shade tolerant than native species (Fig.3b; Table5). Interestingly, the interaction between introduction and weed status was significant for one trait – fruits that persist on the plant (Table5). Native weeds are more likely to exhibit fruits that persist on the plant compared with native nonweeds, whereas introduced weeds are less likely than introduced nonweeds to have fruits that persist (Fig.4a,b).

**Table 5:** The results of logistic regressions testing whether introduced or weedy species are more likely to exhibit each of the following traits, along with the interaction between introduction status and weed status

Trait	Introduction status		Weed status		Introduction × weed	
	χ^2^	*P*	χ^2^	*P*	χ^2^	*P*
Annual	4.00	0.047	46.00	1.20E-11	0.54	0.46
Fast growth rate	28.80	8.10E-08	44.30	2.80E-11	0.27	0.61
High fruit abundance	17.40	3.10E-05	8.00	0.0046	2.10	0.15
Fruit persistence	35.10	3.10E-09	9.80	0.0018	24.10	9.10E-07
Rapid seed spread	0.36	0.55	24.70	6.60E-07	0.12	0.72
Seedling vigor	5.60	0.018	19.50	1.00E-05	2.60	0.1
Vegetative spread	1.20	0.27	29.50	5.70E-08	0.33	0.56
High CaCO_3_ tolerance	1.10	0.29	7.50	0.0063	3.10	0.077
CaCO_3_ tolerance	25.30	4.90E-07	0.73	0.39	2.30	0.13
High salt tolerance	0.01	0.93	2.40	0.12	1.40	0.24
Salt tolerance	27.10	2.00E-07	15.30	9.00E-05	0.19	0.66
Shade tolerance	18.50	1.70E-05	23.20	1.50E-06	0.04	0.84

χ^2^ and *P*-values for each test are presented, and log-odds ratios for the main effects are presented in Fig.3.

Other traits that may distinguish species at different stages of the continuum are CaCO_3_, salt and shade tolerance, as well as vegetative spread. For example, introduced weeds are more likely than introduced nonweeds to exhibit high salt tolerance, whereas there is no significant difference in the likelihood that native weeds and native nonweeds will exhibit this trait. Native weeds are less likely to be shade tolerant but more likely to spread by vegetative means and exhibit high CaCO_3_ tolerance than native nonweeds – in comparison, these traits did not differ between introduced weeds and introduced nonweeds. Thus, some abiotic tolerance and life-history traits appear to be specific to either introduced or native weeds.

We also identified differences between introduced and native weeds. While four of the 12 traits that we tested – annual life form, high growth rate, high fruit abundance, and high seedling vigor (Figs3 and 4) – are consistently more likely to be associated with weedy plants regardless of introduction status, they are also more likely to occur among introduced weeds than native weeds (Fig.4c). We further found that introduced weeds are less likely to be shade and CaCO_3_ tolerant than native weeds (Fig.4c), but are more likely to be both salt and highly salt tolerant (Fig.4c).

#### Genome size and introduction date

We found that the average genome size of weed species is 47% smaller than that of nonweed species (3322.27 versus 6249.15 Mbp, respectively); this difference is highly significant (*P* = 7.58E-06; Table S5). Introduced species have smaller genomes compared with native species (4006.72 and 6665.33 Mbp, respectively; *P* = 3.79E-06). Further, we found a significant interaction between introduction and weed status (*P* = 0.018; Table S5). The genomes of native weeds are *c*. 50% smaller than those of native nonweeds (χ^2^ = 14.52; df = 1; *P* = 0.0001), and although not significantly different (χ^2^ = 1.78; df = 1; *P* = 0.182), the genome size of introduced weeds is on average 35% smaller than that of introduced nonweeds.

**Figure 5:**
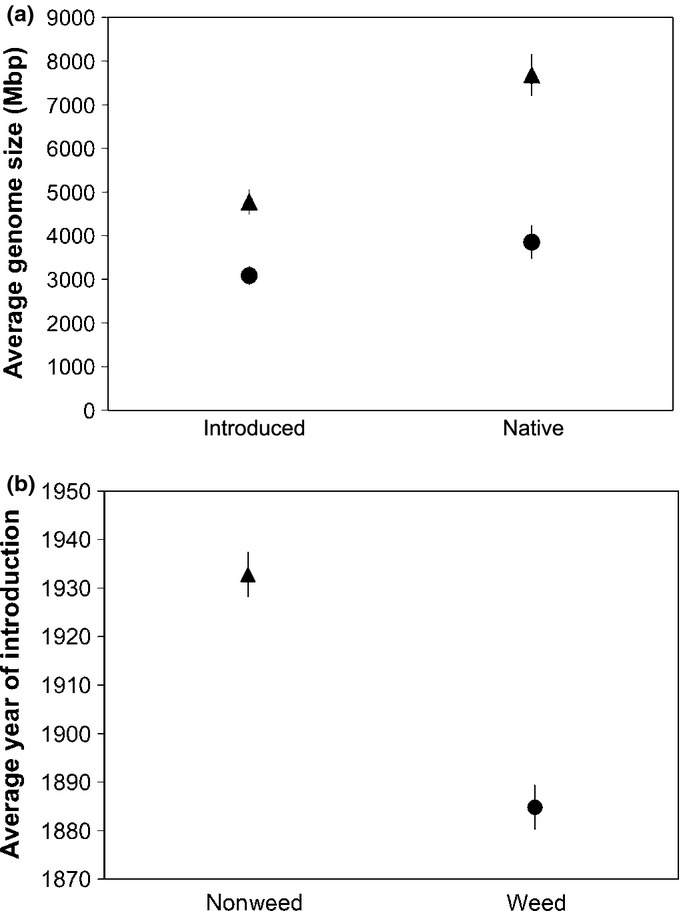
Genome size and introduction date for US plant species. (a) Average genome size estimates (Mbp ± SE) for introduced and native species that are nonweeds (triangle) and weeds (circle). C-values were obtained from the Kew database (http://www.kew.org). (b). Average year of introduction (± SE) (using average first year of herbarium record) for 100 randomly selected introduced nonweed and weed species. Introduction dates were obtained from the Global Information Biodiversity Information facility (http://data.gbif.org/).

Finally, we found that introduced species that are currently considered weedy were introduced to the USA earlier than nonweedy introduced species (mean weedy plant introduction date = 1885; mean nonweedy introduction date = 1933; *P* < 0.001; Fig.5b).

### Herbicide resistance patterns

The majority of herbicide-resistant species in our database – 48 of 67 species (71.6%) – are introduced weeds. We found one introduced nonweedy species (1.5%), 16 native weeds (23.9%), and two nonweedy natives (*c*. 3%) to be herbicide resistant (Fig.2). As would be expected, *c*. 95% of species that are herbicide resistant are also considered weeds – whether native or introduced. Furthermore, we found that only 18% of herbicide-resistant species are natural area invaders (Table S6), whereas the majority of herbicide-resistant species are agricultural weeds – that is, 58% of herbicide-resistant plants are introduced agricultural weeds and 24% are native weeds (Table S6). Our taxonomic selectivity analysis uncovered three genera (*Amaranthus*, *Echinochloa*, and *Lolium*) and nine families (Cyperaceae, Asteraceae, Polygonaceae, Brassicaceae, Malvaceae, Chenopodiaceae, Solanaceae, Poaceae, and Amaranthaceae) enriched for herbicide-resistant species (Table6). All nine families that were overrepresented by herbicide-resistant species were likewise overrepresented by weedy species (Tables1 &). Furthermore, we found that herbicide-resistant weeds, regardless of introduction status, have 33% smaller genomes than nonresistant weeds, although this difference is not significant (average genome size of herbicide-resistant and -nonresistant weeds 2259.26 versus 3386.07 Mbp, respectively; χ^2^ = 0.396; df = 1; *P* = 0.529).

**Table 6:** Plant families in the 2010 US Department of Agriculture (USDA) Plants Database that were more likely to contain herbicide-resistant species than expected by chance, out of 270 families in total

Family	Resistance	No. of species per family	No. of species resistant	Proportion resistant	*R*-value
Cyperaceae	Single	848	3	0.004	3.21E-07
Asteraceae	Multiple	2553	11	0.004	3.41E-39
Polygonaceae	Single	418	2	0.005	1.45E-06
Brassicaceae	Single	649	5	0.008	4.68E-16
Malvaceae	Single	215	2	0.009	2.95E-06
Poaceae	Multiple	1333	22	0.017	1.34E-81
Solanaceae	Single	174	3	0.017	3.99E-09
Chenopodiaceae	Multiple	198	5	0.025	3.23E-12
Amaranthaceae	Multiple	86	6	0.07	6.59E-07

Taxonomically selected families (Family), whether single or multiple resistances were found within the family (Resistance), the total number of species per family in the data set (No. of species per family), the number of resistant species per family (No. of species resistant), the proportion of species that were resistant per genus (Proportion resistant), and the probability of being herbicide resistant (*R*-value) are shown. The expected proportion of herbicide-resistant species from the entire data set was 0.00349.

## Discussion

In this analysis of US plants, we uncovered genera that are more likely to be overrepresented by weedy and introduced species than expected by chance, as well as plant families that are overrepresented by herbicide-resistant species. In line with previous work, we found particular traits, generally related to rapid life cycle and high reproductive rate, that are more likely to be associated with weedy plants than with nonweedy plants, regardless of species’ origins. We also uncovered stress tolerance traits that are more likely to be associated with either native or introduced weeds in comparison to native or introduced nonweeds, suggesting that native and introduced weeds may have different stress adaptations underlying their success. Furthermore, weedy plants in the USA have significantly smaller genomes than nonweeds; this is especially true for natives. We also found that introduced weeds have been present in the USA for *c*. 50 yr longer than have introduced nonweeds. These results support previously discovered trends for weedy and invasive plants from other floras, provide a detailed view of the differences between introduced weeds and native weeds, and provide a comprehensive survey of trends across weedy/invasive plant species within the USA.

The results of our taxonomic enrichment analyses are broadly congruent with global data sets (Daehler, 1998; Pyšek, 1998) and regional floras (Cadotte & Lovett-Doust, 2001); while we found that weedy species are present in all plant families (Barrett, 1992), certain plant genera are more likely to contain weedy and introduced species than other genera. Such assessments are of interest to biological conservation because closely related species probably share similar traits that may underlie weediness or invasiveness, and so taxonomic patterns could provide a useful surrogate for species’ invasive potential (Lockwood, 1999). The ability to predict future invasivity has been a long-standing goal of ecologists (Kolar & Lodge, 2001). Globally, certain plant species are being introduced and expanding in range whereas endemic plant species are going extinct at a high rate, effectively leading to a reduction and/or shuffling of the earth’s biodiversity (Gilbert & Levine, 2013); such changes have been found to significantly impact ecological processes and ecosystem functioning (Vilà *et al*., 2011). The results presented here are not predictive *per se* – and, in fact, it is striking that our weedy genera exhibit a broad diversity of life history, growth form, and mating systems and even range from herbaceous species to grasses and trees. However, that we have identified a relatively small fraction of genera in our total database to be enriched for weedy and/or introduced species (0.64 and 1.41%, respectively) provides conservationists and land managers with a manageable list of plant groups that deserve increased and continued scrutiny. The genera that are overrepresented by both weeds and introduced species – *Polygonum*, *Solanum*, *Acacia*, *Rumex*, *Centurea*, *Urochloa* and *Brassica* – are all groups that deserve such scrutiny if future introductions are planned (e.g. horticulture) and/or if a member of this group is being considered as a possible biofuel or cover crop, or for animal forage.

Furthermore, the identification of genera that are overrepresented by introduced species does not specifically address *where* in the continuum of invasiveness or weediness such species may be positioned – for example, a genus that we identify as enriched for introductions could presumably have many naturalized and self-sustaining species with few of them having attained the status of a plant weed or invasive. Thus, we are specifically interested in which fraction of the US flora is introduced and nonweedy versus introduced and weedy, and if there are particular plant characteristics that differentiate the two. Of the introduced and weedy species in the data set, we found far more species that are introduced and naturalized, but not yet considered weedy (49%), compared with species that are introduced and weedy (29%). The larger fraction of introduced nonweed species compared with introduced weeds should be cause for concern, as introduced species generally take *c*. 50 yr before becoming weedy, that is, the ‘sleeper weed’ effect (Groves, 1999); we note, however, that all introduced plants will not necessarily evolve into becoming weedy or problematic (Davis, 2003). The data we present support the sleeper weed idea – on average, the first herbarium record for weedy introduced species dates to 1882, compared with the first herbarium record for nonweedy but naturalized species which dates to an average of 1931. The lag time in becoming weedy is probably associated with the capability of a population to increase its population sizes to the point of becoming a nuisance (Booth *et al*., 2004) – often a result of particular environmental stimuli, such as habitat disturbances (De Wet & Harlan, 1975), and/or adaptive evolution (Ellstrand, 2009; Schierenbeck & Ellstrand, 2009; Ellstrand *et al*., 2010). This lag time is not always apparent, however, as Carpenter & Cappucino (2005) found no association of introduction time and the extent of plant invasiveness in Canadian flora.

A central theme in invasion ecology since its emergence as a field has been the identification of traits that may predispose species to invasivity (reviewed in Richardson & Pyšek, 2006) – a direction probably influenced by Baker’s list of weedy plant traits (Baker, 1965). Recent criticism of such analyses posits that invasive plant traits are shared by all successful plants and are not necessarily restricted to invasives (Thompson & Davis, 2011) – that is, introduced species that have expanding ranges exhibit the same traits as expanding native species (Thompson *et al*., 1995). Our analysis, in which we contrasted the traits of plants that span the introduced species continuum, as well as native weeds and native nonweeds, provides a more nuanced view: weedy species, regardless of origin, are more likely to exhibit the traits of successful plants as identified above and in previous work (annual life form, rapid growth, high fruit abundance, and high seedling vigor; reviewed in Pyšek & Richardson, 2007; Cousens, 2008). However, we found that introduced weeds are more likely to exhibit these traits than native weeds. In addition, stress tolerance traits such as high salt and calcium carbonate tolerance appear to be specific to either introduced or native weeds, but not both, when compared to nonweeds. These findings represent novel additions to the examination of traits that differentiate weeds from nonweeds and highlight the differences between native and introduced weeds.

We do not have the data to determine whether introduced and native species became weedy following adaptation to salt and CaCO_3_ environments, or if they happened to be pre-adapted to these particular stresses; however, salt and calcium carbonate tolerances would certainly allow species to expand in range whether along coastal regions (e.g. high salt tolerance) or in highly disturbed areas such as agricultural land or roadsides. Calcium carbonate, the main component of agricultural lime, is regularly added to soils to increase pH, and it is naturally found in shallow soils on limestone bedrock. Soils with high pH generally have low micronutrient availability; thus, native species that become weedy may be those that can grow rapidly in low-nutrient soils.

Finally, we found that native weeds are more likely to exhibit fruits that persist on the plant compared with native nonweeds. Introduced weeds, in comparison, are *less* likely to exhibit fruits that persist on the plant compared with introduced nonweeds. This result is in line with comparisons of invasive plants in the floras of Denmark and Ontario which found that invasives are less likely to be dispersed by animals than native species (Andersen, 1995; Cadotte & Lovett-Doust, 2001). Despite the interesting phenotypic comparisons we make and present here, we recognize that there are some limitations of the data. First, the phenotypic traits we examined are binary and presented as ‘presence/absence’ in the USDA Characteristics database; continuous data on these traits, and information on their relative phenotypic plasticity, cannot be assessed here. Furthermore, our analysis does not correct for phylogeny, and thus we cannot distinguish the relative importance of the traits versus the species that exhibit them. As has been noted, phylogenetic corrections may or may not provide different results (Harvey *et al.,* 1995); interestingly, similar traits of invasive plants have been identified using phylogenetic corrections when compared with studies, like ours, that do not control for phylogeny (Pyšek & Richardson, 2007).

Genome size is another trait that can delineate weedy and introduced plants from nonweeds and natives. For example, the average genome sizes of introduced plants are 34% smaller than those of native species, and weeds have smaller genomes than nonweeds. Previous work has uncovered a negative relationship between plant C-values and weediness ‘score,’ in that genome size decreases as the severity of the weed increases (Chen *et al*., 2010). How these genome size differences may contribute to the development of weediness remains experimentally untested. A particularly attractive hypothesis is the idea that genome size may be a primary indicator of weediness (Rejmánek *et al*., 2006), as plants with small genomes often have short generation times, small seeds, and high relative growth rates relative to species with larger genomes (Bennett 1987). The weedy lifestyle may be made possible by small genomes, or small genomes may have been selected over time in plants exposed to new or stressful environments, as plant stress has been shown to select for fast reproduction and a weedy lifestyle (Stanton *et al*., 2000). The smaller genomes of introduced species compared with native species overall suggest that these traits associated with small genomes may also increase naturalization success.

Finally, one of the major aims of this broad analysis was to assess the prevalence of herbicide resistance among weedy and invasive plants. We found that *c*. 73% of herbicide-resistant species in the US flora are introduced weeds whereas 24% of resistant species are native agricultural weeds. In comparison, we identified only 18% of the herbicide-resistant species in the USA to be invaders of natural areas. We also found nine plant families that are more likely to contain herbicide-resistant species than other families. A recent study assessing the potential for taxonomic enrichment of evolved herbicide resistance found three families to be overrepresented by herbicide-resistant members – the Amaranthaceae, Brassicaceae, and Poaceae, which our analyses also pinpointed (Holt *et al.,* 2013). That we uncovered more families enriched for herbicide resistance may be because of differing methodologies. To determine if plant families were overrepresented by herbicide-resistant members, the Holt *et al*. study compared the percentage of resistance within the top 10 most herbicide-resistant families to the percentage of resistance within families with the highest proportion of weeds, as determined using a list of species designated as ‘The World’s Worst Weeds’ (Holm *et al*., 1977, 1979). In contrast, we assessed the chance that a family would be resistant across all species within the data set, and thus our expectation of herbicide resistance should be much lower then that of the Holt *et al*. study.

Regardless, all of the families we detected as enriched for herbicide-resistant species also contain domesticated species, and some contain species that were cultivated by Native Americans for cereals and leaf vegetables (e.g. *Amaranth* and *Chenopodium* spp.) (Dekker, 2011). It is likely that these plant groups have had a long association with agriculture – perhaps historically as crops and currently as crop weeds – and because of this association with agricultural regimes they have been the targets of many herbicide applications. While we do not have the data to determine if the introduced herbicide-resistant species evolved resistance in their native range or within the USA post-introduction, a case of herbicide resistance is only considered as such in weed science if a researcher has noted the development of an herbicide-resistant population from a previously susceptible population (Heap, 2013). Thus, our sample may well be representative of post-introduction herbicide resistance development in the USA.

### Conclusions

The results presented herein provide a broad view of the differences between species in the US flora at differing stages of the continuum and expand upon previous examinations that have focused primarily on introduced weeds – that is, the many studies that have identified the characteristics of the ideal ‘invader’ (reviewed in Pyšek & Richardson, 2007). The novel finding that most herbicide-resistant species within the USA are introduced weeds might be explained by another novel result of this work. We show that introduced weeds are more likely than native weeds to exhibit high fruit abundance, fast growth rate, and high seedling vigor, which are characteristics of r-selected species, or those that allocate towards abundant reproduction. Thus, introduced species that reproduce often, produce a large number of offspring, and inhabit agricultural fields should be viewed with high scrutiny for potential herbicide resistance. Furthermore, we identified differences in stress tolerance between introduced and native weeds – such differences have not, to our knowledge, been reported in the literature.

There are important caveats to the trends that we present. First, we have not assessed species that are introduced but not yet naturalized – thus, our data are restricted to introduced species that are either noticeable or problematic. Secondly, we have no quantitative data on population sizes or ranges of the weedy species within our data set. The presence of an introduced or native weed in our data set does not indicate its severity – it is possible that very few of the weeds in the WSSA list are considered the most noxious or those that lead to the most economic decline. To take this into account, a metric of severity for each of the weeds listed in the database – whether from a vegetation survey, Geographical Information System records, or species presence/absence per county – should be examined. We also have not explicitly determined the ecological conditions under which species may become weedy. Both are certainly influential factors that underlie the emergence of plant weeds. Here we identified plant genera and traits to inform land managers, conservationists, and ecologists of particular plant groups that deserve increased scrutiny, with the aim of stimulating hypotheses about the factors that drive transitions between stages of the weedy plant continuum.

## Supporting Information

**Table S1** The number of individuals used to address each objective of the presented work **Table S2** The phi correlation coefficients between each trait in the analysis **Table S3** The 10 genera (0.35%) out of the 2829 from the 2010 USDA Plants Database less likely to contain weedy species than expected by chance **Table S4** The 30 genera (1.06%) of the 2829 from the 2010 USDA Plants Database that were less likely to contain introduced species than expected by chance **Table S5** ANOVA table showing the effect of introduction and weed status on genome size **Table S6** The representation of herbicide-resistant species in the USA according to status as an agricultural weed versus an ‘invasive’ or natural area invader
